# South African medical interns’ perspectives on the use of point of care ultrasound

**DOI:** 10.4102/safp.v65i1.5772

**Published:** 2023-12-26

**Authors:** Pierre-Andre Mans, Oladele V. Adeniyi

**Affiliations:** 1 Department of Family Medicine, Faculty of Health Sciences, Cecilia Makiwane Hospital, Walter Sisulu University, East London, South Africa

**Keywords:** medical interns, internship training, point of care ultrasound, undergraduate ultrasound training, postgraduate ultrasound training

## Abstract

**Background:**

Point of care ultrasound (POCUS) has become such a part of patient care that it is included in undergraduate medical training in many high-income countries. In South Africa, despite the availability of ultrasound units, there is no information on the exposure or training required for medical interns to perform POCUS in their community service year. This study examines interns’ self-reported POCUS training and competency, as well as their perceived readiness for their community service year.

**Methods:**

In this cross-sectional web-based survey, 43 interns were invited to complete a self-administered questionnaire after completing their 6-month decentralised family medicine rotation in 2022.

**Results:**

Thirty complete responses (69% response rate) were included for analysis. Eleven graduates from three medical schools reported undergraduate exposure to POCUS. Ten participants completed formal postgraduate ultrasound training. Eight participants felt confident to independently perform POCUS. Thirteen participants felt adequately prepared for their community service year, 10 of whom had received POCUS training. Nearly all the participants (29 of 30) felt that ultrasound training should be incorporated into both undergraduate and internship training.

**Conclusion:**

Medical interns expressed a need for more POCUS training. Most of the studied sample did not feel adequately prepared to perform POCUS independently. The high uptake of additional ultrasound courses highlights the need to include POCUS training. More research is needed to determine the extent and level at which POCUS training should be offered to medical interns in the South African setting.

**Contribution:**

This study looks at the perceived need of South African medical interns for formal POCUS training. It highlights a potential gap in training based on the expected clinical requirements of the community service year.

## Introduction

Point of care ultrasound (POCUS) has become an integral component of patient evaluation in almost all fields of medicine.^1^ Like a stethoscope, POCUS uses sound waves to guide clinician decision making and is described as the stethoscope of the future.^2^ There is an increasing trend in the uptake of POCUS among doctors owing to its ease of access and perceived benefit in patient outcomes – even though there is limited evidence that POCUS improves patient outcomes.^3^ The South African ideal hospital framework describes the placement of ultrasound units in both the emergency and labour ward of district hospitals.^4^ The South African maternity care guideline also describes the use of ultrasound as part of the routine care at district hospital level.^5^ There is clearly an expectation of ultrasound use and its central role in the provision of quality of healthcare service delivery at district hospital level.

The South African undergraduate medical school and 2-year internship training are designed to prepare medical doctors for the generalist work they are expected to perform during their community service year.^6^ Yet there is no formal national curriculum or minimum competencies for POCUS use in the South African context. There is a clear knowledge gap with regard to what ultrasound skills should be available in South African district hospitals, and to what level these skills should be trained.

It should be noted that POCUS training has been incorporated into undergraduate medical programmes in some high-income countries.^7,8^ However, a recent Saudi Arabian study found that only 22% of medical interns received undergraduate POCUS training, while the majority practising POCUS were self-taught.^9^ Because medical interns are identifying the need and facilitating their own learning of POCUS, understanding their learning needs may be a key component to standardising their training.^10^ This study examines the medical interns’ self-reported POCUS training and competency, as well as their perceived readiness for community service at district hospitals in South Africa.

## Methods

This cross-sectional study was conducted on a group of second-year medical interns in the East London Hospital Complex (Frere and Cecilia Makiwane Hospitals). The study did not include interns from other training sites because the POCUS training exposures may have been different across sites. In the East London Hospital Complex, informal POCUS exposure and training is routinely offered in Obstetrics and Gynaecology, Emergency Medicine, and family medicine. However, this training is largely unstructured across the various departments. The survey was done after the interns had completed their 6-month decentralised family medicine rotation, in their second year of training. At this point, all participating interns had completed 18 months of training, with only the 2-month rotations in Orthopaedics, Psychiatry and Anaesthetics each remaining. All of the interns had completed the informal POCUS exposure in their Obstetrics and Gynaecology, Emergency Medicine and family medicine rotations. The decentralised family medicine rotation also required that all the interns spend 2 months in one of the four accredited district hospitals in the central region of the Eastern Cape province. Census method of data collection was implemented to allow for inclusivity of all interns who had experienced the decentralised family medicine rotation in the East London Hospital Complex.

The study was conducted using a web-based survey tool (Welphi, Lisbon, Portugal) with written online consent and anonymised responses. The survey consisted of six validated binary questions (yes/no) with respondent options (Appendix 1). The survey link was emailed to all of the interns on 24 August 2022. Additional phone calls were made to interns who did not respond within 2 weeks in order to enhance response rate. Participants were reminded weekly until they had completed the survey or elected to withdraw from the study.

## Results

**FIGURE 1:**
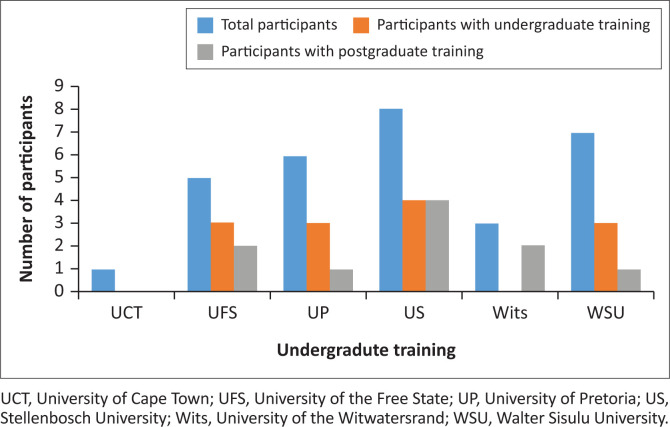
Bar chart showing the undergraduate and postgraduate exposure of medical interns to point of care ultrasound.

Of the 43 medical interns who completed the 6-month decentralised family medicine rotation, 30 completed the online survey (69%). The participants had graduated from six medical schools, with only three of the medical schools offering some form of undergraduate ultrasound training (Figure 1).

Of the total participants (*n* = 30), eight had completed the Emergency Medicine Society of South Africa (EMSSA) POCUS core course,^11^ and two had completed other short courses on ultrasound. Thus, 10 of the participants (33.3%) had pursued some formal postgraduate ultrasound training. The association between undergraduate and postgraduate ultrasound training is graphically represented in Figure 1. The correlation of these non-linear variables was calculated using Spearman correlation coefficient, with the resulting Spearman’s Rho indicating a strong correlation at 0.688.

Of the total participants (*n* = 30), only eight (26.7%) felt comfortable with independently using ultrasound as part of patient evaluation in their community service. All the participants who felt comfortable (*n* = 8) had received some form of ultrasound training, either undergraduate, postgraduate or both.

When asked where POCUS training should be offered, all but one participant (96.7%) said that POCUS training should be incorporated into the undergraduate medical programme. In addition, all but one medical intern (96.7%) felt that more POCUS training should also be included during the internship period.

The final question gauged the interns’ perceived readiness for their community service year. Only 13 of the 30 participants felt ready for their community service year – implying that a slight majority of the medical interns (56.7%) did not feel adequately prepared. Among those who felt adequately prepared for their community service year (*n* = 13), 10 had received POCUS training. Only three participants reported receiving both undergraduate and postgraduate POCUS training. All three were confident about their readiness for community service. The summary of findings is presented in Table 1.

**TABLE 1:** Breakdown of responses to ultrasound training.

Positive responses recorded	All participants (*n* = 30)		Undergraduate Training (*n* = 11)		Postgraduate Training (*n* = 10)		No Formal Training (*n* = 12)	
	*n*	%	*n*	%	*n*	%	*n*	%
Do you feel adequately prepared for your community service year?	13	43	7	64	6	60	3	25
Do you feel comfortable to independently use an ultrasound machine in your community service year?	8	27	5	45	5	50	0	0
Do you feel more ultrasound training is required in internship training?	29	97	10	91	10	100	12	100
Do you feel more ultrasound training is required in undergraduate training?	29	97	10	91	10	100	12	100

## Discussion

Currently, no research has examined the perceived readiness and confidence of medical interns in performing POCUS during their community service year in South Africa. This study aimed to bridge this gap by offering some insights into interns’ perspectives on their perceived needs for POCUS training, and their readiness for their community service year. It is noteworthy that three of the medical schools represented did provide some undergraduate exposure to their students. Yet undergraduate POCUS training was not reported by all graduates from these universities. This leaves the question as to what extent POCUS training was offered – whether it was informal bedside teaching or formal structured teaching. It is unclear if there is a structured curriculum for training POCUS in these medical schools.

The fact that a third of medical interns (33.3%) were willing to purchase formal POCUS training within 18 months of graduating confirms the significance of their perceived training need. The EMSSA POCUS core course was the most attended formal training in this study sample. It is a 1-day exposure to basic ultrasound physics and knobology, as well as the basic POCUS examinations used in emergency medicine. Following this course, the participants need to complete a recommended number of supervised scans in a clinical environment, whereafter the participant may sit a credentialling assessment.^10^

With only eight interns feeling comfortable to independently use ultrasound, it is not surprising that nearly all (96.7%) felt that POCUS training should be introduced at both undergraduate and internship levels. This has become the standard of practice in some high-income countries.^8^ The low level of perceived readiness for community service reported by 13 of the respondents should be interpreted with caution. The low number of participants and limited number of universities represented may have influenced this number. In addition, the finding is affected by the participants’ limited clinical exposure in the 2 years prior to the study as a result of restrictions arising from the coronavirus disease 2019 (COVID-19) pandemic. Other studies have reported that up to 13% of interns did not have the required amount of clinical exposure.^12^ This may reflect site-specific training gaps or a much bigger problem in medical internship training. These and other factors may all have contributed to the low level of perceived readiness in this sample of medical interns. However, the significant association between ultrasound training and perceived readiness for community service and independent ultrasound use is noteworthy and warrants further research.

A concern regarding interns’ readiness for community service was, however, observed in a recent study looking at simulation-based assessments of interns’ management of failed obstetric intubation, where only 40% passed the assessment.^13^ Additional research is needed to clarify this observation. Of interest is the response to the question of when ultrasound training should be introduced; 96% of the participants recommended that POCUS training be included at both undergraduate level and in the internship years. The authors expected that participants would choose either of these options, probably the latter, but not both. However, their suggestion is probably the correct way of approaching ultrasound education, with the World Federation of Ultrasound in Medicine having stated that ultrasound training should be seen as longitudinal learning, possibly a life-long journey.^8^

While this study provides insightful information on POCUS training needs and readiness for the community service year, the limitations of the study cannot be ignored. This study does not have adequate representation of all South African medical schools, nor a representative sample of medical interns from different training sites in the country to make widespread conclusions. Given that these intern doctors are yet to complete their clinical rotations in anaesthesia, Orthopaedic and Psychiatry, it is unclear how these domains would have influenced the findings. It should also be noted that this study focused solely on POCUS skills and its implication for service delivery at the district hospital level. Whether the interns attended other short courses, surgical skills, essential steps in the management of obstetric emergencies, basic life support and many others were not examined. As such, it is unable to gauge how these additional skills or lack thereof influence the perceived readiness of the interns for their community service. In addition, the use of Likert scale with five respondent options could have expanded the responses of the interns on the item measures rather than a binary option. More studies are needed on the POCUS training needs of medical interns and practitioners in the country.

## Conclusion

In keeping with international trends, South African trained medical interns expressed a need for more POCUS training. The majority of the studied sample did not feel adequately prepared to perform ultrasound evaluations independently, nor did they feel adequately prepared for their community service year. This is a concerning finding, and further research that is adequately broad in scope is needed to investigate it. The high uptake of additional ultrasound courses highlights the need to include POCUS into medical training. More research is needed to determine the extent and level at which POCUS training should be offered in the South African setting.

## Appendix 1

### Intern POCUS survey questions

1. Did you receive any undergraduate ultrasound training?
2. Do you have any formal training in ultrasound?
3. Do you feel adequately prepared for your community service year?
4. Do you feel comfortable to independently use an ultrasound machine in your community service year?
5. Do you feel more formal ultrasound training is required during your internship?
6. Do you feel more formal ultrasound training is required during undergraduate training?
